# Utilization of donor CLL-1 CAR-T cells for the treatment of relapsed of acute myeloid leukemia following allogeneic hematopoietic stem cell transplantation

**DOI:** 10.3389/fimmu.2024.1491341

**Published:** 2025-01-17

**Authors:** Meng Zhang, Xiaomei Zhang, Jiaxi Wang, Wenyi Lu, Xia Xiao, Hairong Lyu, Xiaoyuan He, Yedi Pu, Juanxia Meng, Cuicui Lyu, Xinping Cao, Mingfeng Zhao

**Affiliations:** ^1^ Hematology Center, National Key Clinical Discipline of Pediatric Hematology, National Key Discipline of Pediatrics (Capital Medical University), Key Laboratory of Major Diseases in Children, Ministry of Education; Beijing Children’s Hospital, Capital Medical University, National Center for Children’s Health, Beijing, China; ^2^ Department of Hematology, Tianjin First Central Hospital, Tianjin, China; ^3^ School of Medicine, Nankai University, Tianjin, China

**Keywords:** CLL-1, CAR-T, AML, Donor-derived, HSCT

## Abstract

**Background:**

Human C-type lectin-like molecule 1 (CLL-1) represents a promising therapeutic target for Chimeric antigen receptor T (CAR-T) cells therapy in the treatment of acute myeloid leukemia (AML). In this study, we aimed to evaluate the efficacy and safety profile of donor-derived CLL-1 CAR-T cells in AML patients who experienced relapsed post-transplantation.

**Methods:**

14 AML patients who experienced relapse following allogeneic HSCT were enrolled in our clinical trial. However, 2 patients withdrew from the study due to rapid disease progression. 12 participants received donor-derived CLL-1 CAR-T cells and were categorized into 3 groups based on the dosage of infused CAR-T cells dose (Group A:0.5×10^6^/kg, Group B:1×10^6^/kg, Group C:1.5×10^6^/kg). And scRNA-seq was used to reveal CLL-1 CAR-T cells dynamics in a CAR-T cells infusion products and PBMCs at the peak of expansion for patient 4.

**Results:**

CLL-1 CAR-T cells were well tolerated by all 12 patients. Cytokine Release Syndrome (CRS) was observed in all patients, with 5 patients experiencing grade ≥3. 3 patients developed cytokine release syndrome-associated encephalopathy (CRES), and 1 patient had a grade 4 severity level. All patients demonstrated a reduction in tumor burden, while 7 patients (58.33%) achieved MRD-CR and 2 patients (16.67%) reached MRD+CR. CAR-T cells expansion was detectable in all 12 patients, with the median time of peak expansion was 9 days (range: 7-11 days). In patient 4, compared to the pre-reinfusion state, CD4+ cells at the peak of expansion showed upregulation of cell killing-related genes and memory T cell-related genes (*P* < 0.01).

**Conclusions:**

The CLL-1 CAR-T cells therapy derived from allogeneic donors demonstrates both safety and efficacy in the management of relapsed AML following allogeneic HSCT. And adjusting the ratio of CD4+ CAR-T cells and CD8+ CAR-T cells prior to infusion may help mitigate CAR-T cell-related side effects.

**Clinical trial registration:**

https://www.chictr.org.cn/, identifier ChiCTR2000041054.

## Background

Acute myeloid leukemia (AML) stands as a prevalent hematologic malignancy, presenting a persistent challenge due to high rates of relapse. Hematopoietic stem cell transplantation (HSCT) has emerged as a potential therapeutic approach. However, approximately 40% of AML patients experience relapse after HSCT, resulting in limited treatment options and unfavorable prognosis (1, 2). Currently, the conventional strategies for managing those patients include donor lymphocyte infusion (DLI) or secondary transplantation. Nevertheless, there is no significant difference in overall survival (OS) between the two treatment methods, with a 2-year OS rate remaining around 25%. Therefore, it is crucial to urgently explore novel therapeutic alternatives to address this pressing clinical need (3).

CAR-T cells therapy is a potential approach for the treatment of high-risk AML. Currently, the potential targets include CD33, CD123, NKG2D, FLT3, among others (4–7). Preclinical experiments have demonstrated that CAR-T cells can effectively eliminate AML tumor cells in mice. However, in clinical applications, these targets still face issues such as insufficient therapeutic effects or significant side effects (8).

Human C-type lectin-like molecule 1 (CLL-1) is a type II transmembrane glycoprotein expressed in leukemic stem cells and progenitor cells. CLL-1 CAR-T cells demonstrate remarkable efficacy in eradicating tumor cells in AML mouse models. Moreover, CLL-1 is highly expressed in over 90% of AML patients, thus emerging as a potential therapeutic target (9). 8 children with refractory AML were treated with CLL-1 CAR-T cells, 4 patients achieving complete remission (CR) and 2 patients achieving minimal residual disease (MRD+) (10). Subsequently, we reported the results from our center. 10 adult patients with relapsed/refractory AML using CLL-1 CAR-T cell therapy, 7 patients achieved incomplete hematologic recovery (CRi) or CR (11). These findings highlight the promising therapeutic potential of CLL-1 CAR-T cells therapy for managing AML.

Currently, the safety and efficacy of CLL-1 CAR-T cells in AML patients who have relapsed after HSCT remain uncertain. In such cases, allogeneic CAR-T cells derived from the donor’s T cells could serve as a viable therapeutic alternative. Unlike autologous CAR-T cells, allogeneic CAR-T cells not only target and eliminate tumor cells but also receive activation signals through T cell receptors (TCR), thereby mediating the graft-versus-leukemia (GVL) effect (12). This dual signaling mechanism has the potential to enhance anti-leukemia efficacy. The objective of our study is to evaluate the effectiveness and safety of CLL-1 CAR-T cells in AML patients who have relapsed after HSCT.

## Methods

### Study design and patients

Between January 2021 and September 2023, a total of 14 patients were enrolled in our clinical trial, receiving administration of CLL-1 CAR-T cells (ChiCTR2000041054). The primary inclusion criteria consisted of (1): confirmed diagnosis of AML based on the European Leukemia Net (ELN) guidelines (13); (2) prior HSCT with subsequent relapse; (3) CLL-1 expression in tumor cells exceeding 80%; (4) the donor is able to accept single-cell collection to obtain sufficient T cells; (5) absence of active bacterial, fungal, or viral infections; and finally, (6) capacity to provide informed consent by signing the appropriate documentation. Notably, individuals with a history of malignancy or severe renal, hepatic, or cardiac dysfunction were excluded from participation.

All patients underwent a lymphodepletion regimen comprising cyclophosphamide (500mg/m^2^) and fludarabine (30mg/m^2^) for three days prior to CLL-1 CAR-T cells infusion. Following completion of the lymphodepletion regimen, donor-derived CLL-1 CAR-T cells were administered one day later. Patients were stratified into 3 groups based on the dose of infused CAR-T cells (Group A:0.5×10^6^/kg, Group B:1×10^6^/kg, Group C:1.5×10^6^/kg). Therapeutic response was evaluated at day 14 post-infusion. Upon obtaining patient consent. And second transplantation with a different donor or cryopreserved hematopoietic stem cells boosts (2×10^6^/kg CD34+ cells) were administered. All patients were followed up until disease progression and long-term outcomes were monitored until death.

### Treatment response evaluation

On the 14th day after CAR-T cells infusion, efficacy assessment should be conducted according to the NCCN guidelines (14). CRS was graded based on the criteria of Lee et al (15). In cases of CRS grade ≥2, Tocilizumab administration is recommended for treatment. If symptoms persist, glucocorticoid therapy may be considered as an adjunct. For CRS grade ≥3, concurrent consideration of plasma exchange and Ruxolitinib may be warranted. The severity of cytopenia should be graded following the ASCO guidelines (16).

### Single-cell RNA sequencing

This study used Single-cell RNA sequencing (scRNA-seq) to reveal CLL-1 CAR-T cells dynamics in infusion products and PBMCs with peak amplification. Firstly, scRNA-seq library preparation and sequencing are performed. Then we utilized the SeekOne^®^Tools to preprocess the sequence data and align it with the human GRCH38 reference genome in order to generate a gene expression matrix. To filter out low-quality cells, we employed the seurat package(version 4.1.1), excluding cells with a detected gene count less than 100 or exceeding 30000. Median absolute deviation(MAD) variance normalization was applied to eliminate mitochondrial gene-related effects. Unsupervised cell clustering was carried out using the Seurat package (version 4.1.1). We employed Harmony (version 0.1) to perform principal component (PC) analysis. Utilizing the first 30 PCs, we identified cell clusters at a resolution of 0.8. The outcomes of the cluster analysis were graphically represented using Uniform Manifold Approximation and Projection (UMAP). Cell types were categorized based on their resemblance to the Human Primary Cell Atlas Data, leveraging the Single R package (version 1.8.1). For T cells, a sub-clustering was performed at a resolution of 0.5, with each subcluster annotated based on specific marker genes identified within them. To isolate CAR-T cells from the single-cell clusters, we integrated CAR gene sequences (RQR8 and CLL1 scFv) into the human genome for comparison, facilitating the detection of CAR-T gene expression. Additionally, corresponding metadata examples included tags indicating the detection of CAR-T cells. Finally, the FindAllMarkers modifier function was used to identify the gene. The functional annotation and enrichment analysis of genes were performed utilizing the clusterProfiler package (version 4.2.0).

### Statistical analyses

The present study employed an observational research design. Descriptive statistics were utilized to analyze safety and efficacy, with means accompanied by corresponding standard deviations or medians along with ranges for continuous variable, while frequencies or percentages were used for categorical variables. Graphical representation was generated using the GraphPad Prism software. A significance level of *P*<0.05 denoted statistical significance.

## Results

### Patient characteristics

A total of 14 patients were enrolled and underwent apheresis. However, 2 patients withdrew from the study due to rapid disease progression prior to CAR-T cells infusion. The remaining 12 evaluable patients were included in **Table 1**, comprising of 5 males and 7 females. The median age was 30.5 years (range:10-48 years), and the median number of prior treatment lines were 5.5 (range:3-9 lines). All patients relapsed after HSCT, with patient number 2 and 11 relapsing after second transplantation. Among them, 2 patients (patient 2 and 5) had central leukemia, whereas another 2 individuals (patient 2 and 11) had extramedullary infiltration (kidney and uterus). Additionally, 3 patients demonstrated high-risk cytogenetic and molecular features (patient 4, 6, and 9). The medium time to relapse after allo-HSCT was 133 days (64-765). The median tumor burden was 20.21% (range:3.09%-69.63%). Patients 1 and 4 received chemotherapy combined with DLI post-transplantation, however, both were ineffective.

**Table 1:** Characteristics of patients before CAR-T cell treatment.

ID	Age/Sex	Diagnosis	Fusion gene	Gene mutation	Karyotype	Prior lines of treatment	Central leukemia	Extramedullary invasion	Pre CAR-T disease burden (%)	CLL-1 Positivity (%)	CLL-1 CAR-T cell infused/kg
1	10/M	AML-M2b	AML-ETO, WT1	C-KIT	45, XY, t(8,21)	8	No	No	22.42	98	1.5×10^6^
2	17/F	AML-M4	/	CEBPA	46, XX [20]	4	Yes	Yes	58	92.3	5×10^5^
3	31/M	MDS-EB2	/	IKZF1	46, XY[20], t (3,21)	3	No	No	4.76	95.6	1×10^6^
4	48/F	AML-M5	WT1	SF3B1,GATA2, CBL	46, XX, inv (3) (q21,q26.2) (3)/46, XX (1)	7	No	No	20	94	1×10^6^
5	12/F	AML	NUP98-NSP1, WT1	KRAS,CCND, NRAS, PTPN11	N	7	Yes	No	20	83	1.5×10^6^
6	36/M	MDS-RAEB1	WT1	TP53	5q-	4	No	No	65.54	99.6	1.5×10^6^
7	30/M	AML	/	FLT3-ITD	46, XY [20]	5	No	No	9.1	89	1.5×10^6^
8	47/F	AML	WT1	CEBPA, SETD2, SETBP1,FAT1	/	9	No	No	69.63	82.3	5×10^5^
9	18/F	AML-M2	/	FLT3-ITD, RUNX1	N	6	No	No	7.93	88.92	5×10^5^
10	33/F	AML-M2b	AML1-ETO	/	N	4	No	No	3.09	81	1.5×10^6^
11	29/M	AML-M5	/	/	N	3	No	Yes	49.99	98.5	5×10^5^
12	47/F	AML-M5	N	N	N	8	No	No	22.8	88	1×10^6^

CLL-1, human C-type lectin molecule 1; CAR-T, chimeric antigen receptor T cells; AML, acute myeloid leukemia.

### Efficacy

**Figure 1:**
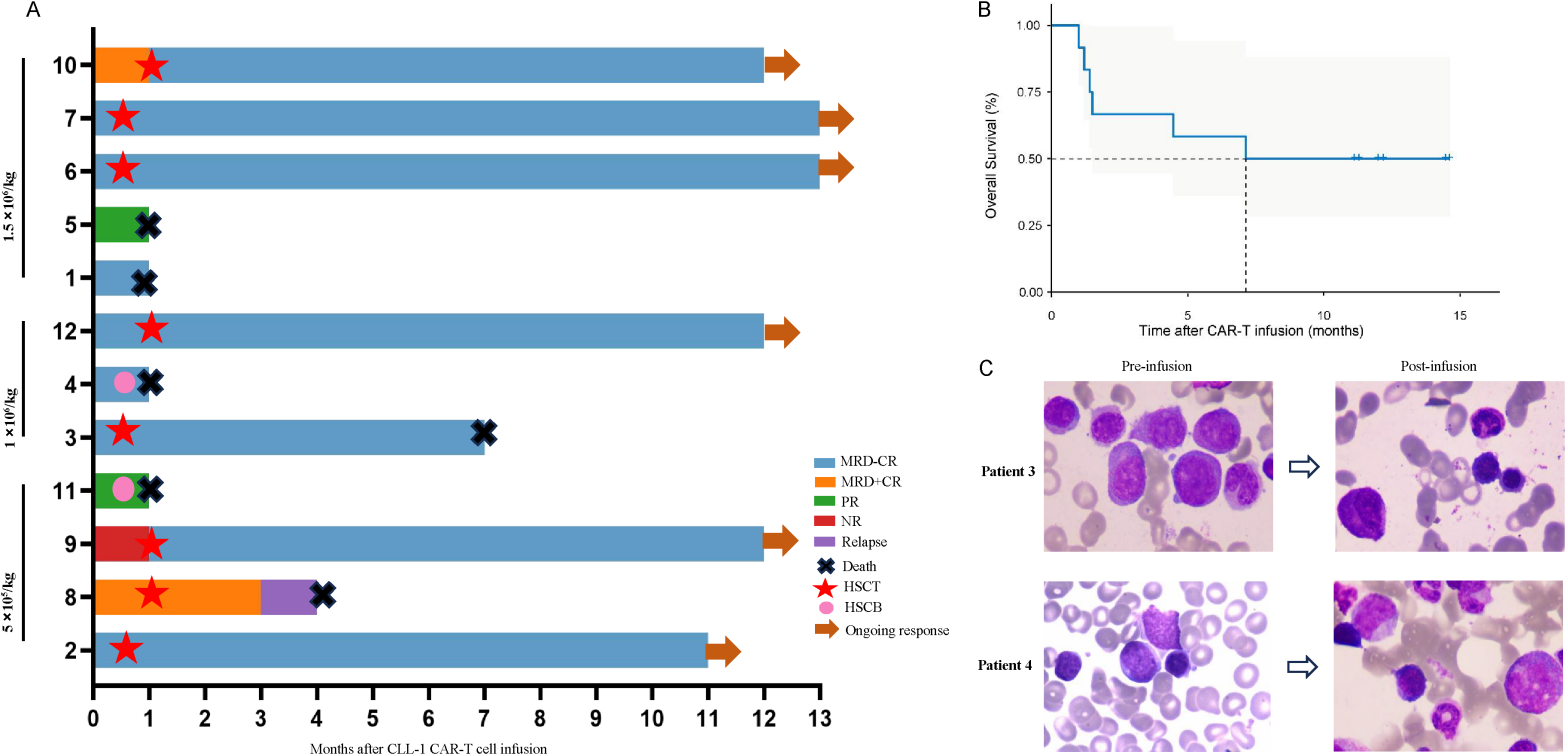
Clinical outcome following secondary HSCT. **(A)** The swimmer plot depicts the clinical response and follow-up individual patients who underwent secondary HSCT after CLL-1 CAR-T cell therapy, represented by distinct colors in the swimmer lanes. **(B)** Kaplan-Meier estimates show the overall survival outcomes in patients undergoing secondary HSCT following CLL-1 CAR-T cell therapy. **(C)** Bone marrow smears of patient 3 and patient 4 before and after infusion.

All 12 patients exhibited a reduction in tumor burden, with 7 patients (58.33%) achieved MRD-CR (1 in Group A, 3 in Group B, and 3 in Group C). 2 patients (16.67%) reached MRD+CR (1 in Group A and the other in Group C) (**Figures 1A, C**). Another 2 patients (16.67%) achieved PR (1 in Group A and the other in Group C). Notably, 1 patient (8.33%) in Group A was NR. The median remission time was 7.13 months in all cohort (**Figure 1B**).

The median follow-up time was 12 months. 8 patients bridging HSCT, with the median time was 22 days (range:9-27) from cells infusion to transplantation. Until the end of follow-up, 2 patients (patient 3 and 8) died due to COVID-19 infection and recurrence of primary disease, respectively. 2 patients (patient 4 and 11) received cryopreserved hematopoietic stem cells on D15 and D23, respectively. Unfortunately, both patients died because of severe CRS and severe infection(Patient 4 received treatment with glucocorticoids, tocilizumab, and plasma exchange, and Patient 11 was treated with glucocorticoids, tocilizumab, and ruxolitinib). Patient 1 and 5 refused HSCT due to financial and other reasons, but died of severe infection and intracranial hemorrhage on D26 and D23 due to prolonged granulocytopenia and thrombocytopenia, respectively (**Figure 1A**).

### Safety

We evaluated the adverse effects associated with CLL-1 CAR-T cells (**Table 2**). CRS occurred in all patients, with 5 patients experienced grade ≥3. 3 patients were from the highest dose group (1.5×10^6^/kg), and 7 cases were from the lower dose group (5 were grade 2 and 2 were grade 1). 3 patients developed CRES, with 1 patient graded as 4. Simultaneously this patient experienced grade 4 CRS and died despite attempts at treatment. Fortunately, the CRS and CRES in the remaining patients were manageable and controllable.

**Table 2:** Patient response and toxicity.

ID	Conditioning regimen	CLL-1 CAR-T cell infused/kg	Transduction rate (%)	Response	CRS grade	CRES grade	Treatment	Additional therapy after CAR-T	Neutrophil Engraftment	Platelets Engraftment	Acute GVHD	Chronic GVHD
1	FC	1.5×10^6^	30.21	MRD-CR	3	1	Tocilizumab Dexamethasone Ruxolitinib	No	/	/	/	/
2	FC	5×10^5^	8.39	MRD-CR	2	0	Dexamethasone	Haplo-HSCT	D15	D16	2	0
3	FC	1×10^6^	48.34	MRD-CR	2	0	Tocilizumab Dexamethasone Ruxolitinib	Haplo-HSCT	D12	D13	1	0
4	FC	1×10^6^	33.96	MRD-CR	4	4	Tocilizumab Methylprednisol Ruxolitinib Plasma exchange	No	/	/	/	/
5	FC	1.5×10^6^	26.68	PR	2	0	/	No	/	/	/	/
6	FC	1.5×10^6^	41.56	MRD-CR	2	0	Dexamethasone	Haplo-HSCT	D23	D11	2	0
7	FC	1.5×10^6^	39.05	MRD-CR	3	0	Tocilizumab Dexamethasone Ruxolitinib	MUDT	D13	D13	0	1
8	FC	5×10^5^	38.22	MRD+CR	3	1	Tocilizumab Dexamethasone Ruxolitinib	Haplo-HSCT	D19	D19	1	1
9	FC	5×10^5^	15.54	NR	1	0	/	Haplo-HSCT	D18	D18	1	1
10	FC	1.5×10^6^	25.02	PR	3	0	Tocilizumab	Haplo-HSCT	D20	D20	2	0
11	FC	5×10^5^	28.21	MRD-CR	1	0	/	No/	/	/	/	/
12	FC	1×10^6^	70.38	MRD+CR	2	0	/	Haplo-HSCT	D14	D15	0	0

CLL-1, human C-type lectin molecule 1; CAR-T, chimeric antigen receptor T cells; CRS, cytokine release syndrome; CRES, cytokine release syndrome-associated encephalopathy; GVHD, graft-versus-host disease; MRD, minimal residual disease; CR, complete response; HSCT, hematopoietic stem cell transplantation; MUDT, HLA- matched unrelated donor transplantation.

**Figure 2:**
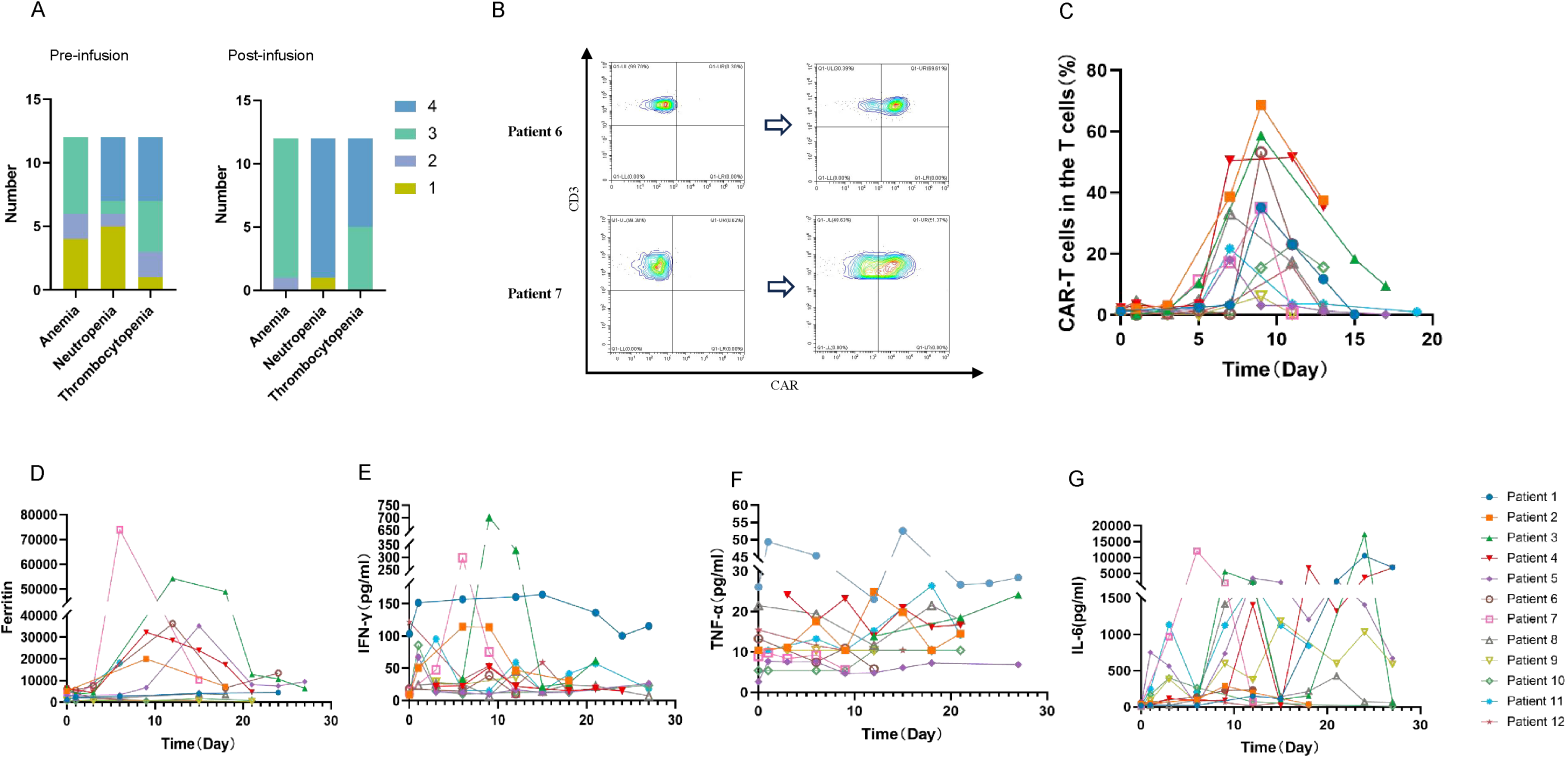
Adverse events and kinetics of peripheral blood biomarkers after CLL-1 CAR-T cell infusion. **(A)** Different levels of cytopenia observed in patients pre and post CLL-1 CAR-T infusion. **(B)** Using the flow cytometry evaluates the peak values of CAR-T cells in patient 6 and 7. **(C)** The ratio of CAR-T cells (CAR-T cells did not specifically distinguish between CD4 and CD8) to T cells in peripheral blood at various time periods. **(D-G)** Peripheral blood serum levels of Ferritin, IFN-γ, TNF-α and IL-6 before and after CAR-T cell infusion.

It’s noteworthy that all patients experienced severe pancytopenia, as shown in **Figure 2A**. 11(91.67%) patients developed grade 4 neutropenia, and 7(58.33%) patients had grade 4 thrombocytopenia. Due to the severe pancytopenia, 8 patients received HSCT with reduced-intensity conditioning regimen and 2 patients received cryopreserved hematopoietic stem cells as boosts. Successful engraftment of neutrophils and platelets was achieved in 8 cases (**Table 2**). Other common adverse effects observed included hypophosphatemia, hypocalcemia, fatigue, elevated liver enzymes, and so on (**Table 3**).

**Table 3:** Adverse events after first infusion of CAR-T cells.

Adverse events	Any n (%)	Worst Grade 3 n (%)	Worst Grade 4 n (%)
Any grade 3 or higher AE	12		
Encephalopathy	2	0	1
Anemia	12	11	0
Neutropenia	12	0	11
Hypoxia	3	1	0
Thrombocytopenia	12	5	7
Acute kidney injury	0	0	0
Aspartate aminotransferase increased	9	0	0
Cardiac failure	1	0	0
Delirium	2	0	0
Fatigue	10	2	8
Hemorrhage intracranial	2	1	0
Hypocalcemia	1	0	0
Hyponatremia	2	0	0
Hypophosphatemia	1	1	0
Hypotension	3	0	1
Metabolic acidosis	1	1	0
Pseudomonal sepsis	3	0	0
Restlessness	2	0	0
Tremor	1	1	0
Urinary tract infection	3	0	0
dedma	1	1	0
Oral herpes	1	0	0

CAR-T, chimeric antigen receptor T cells; AE, adverse events.

### CLL-1 CAR-T cells expansion and persistence in peripheral blood

We evaluated the expansion of CLL-1 CAR-T cells using flow cytometry. As shown in the **Figures 2B, C**, CAR-T cells expansion was detectable in all 12 patients, with the median time of peak expansion was 9 days (range: 7-11 days). Notably, to reduce the side effects, we used Ruxolitinib and steroids to terminate CAR-T cell proliferation before HSCT or donor stem cell support. Consequently, CAR-T cells were undetectable after the third week. Varying degrees of elevation in ferritin and various cytokines were observed after CAR-T cells infusion (**Figures 2D-G**).

### scRNA-seq and clustering analysis of the CAR-T products

**Figure 3:**
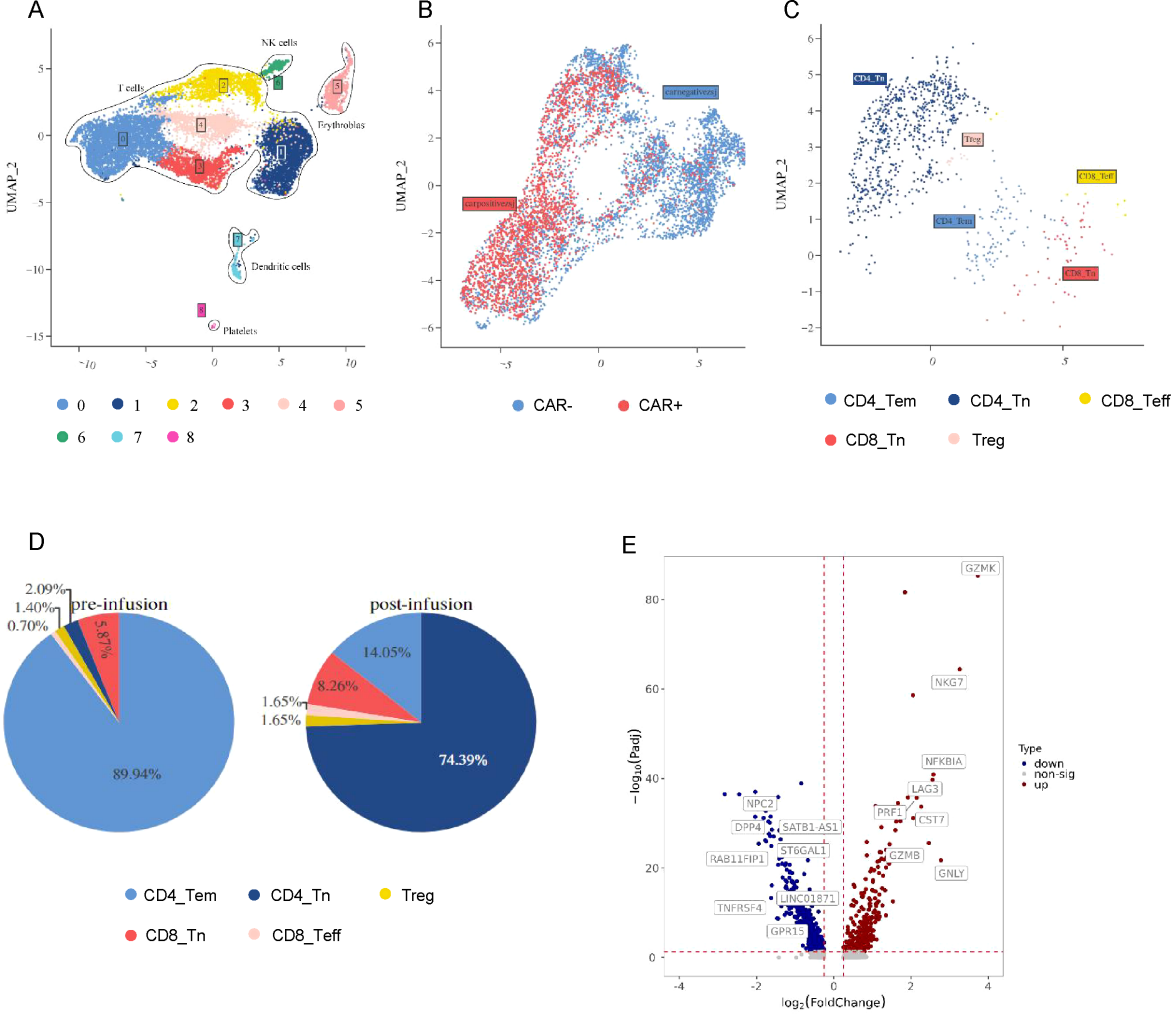
The scRNA-seq landscape in patient 4. **(A)** The distribution of CAR-T product and PBMC after infusion in patient 4 is visualized on the UMAP plot. **(B)** The UMAP plot divided T cells from **(A)** into CAR^pos^ T cells and CAR^neg^ T cells. **(C)** CAR^pos^ T cells were divided from **(B)** and shows 5 clusters. **(D)** The pie chart illustrates the distribution of cell clusters in CAR^pos^ T cells prior to and following treatment. **(E)** The volcano plot illustrates the differential gene expression in CAR^pos^ T cells pre- and post-treatment.

scRNA-seq was used to reveal CLL-1 CAR-T cells dynamics in a CAR-T cells infusion products and PBMCs at the peak of expansion for patient 4. A total of 15,414 cells were captured. After filtering out potential dead cells, UMAP dimensionality reduction and clustering analysis were performed on the filtered cells with good quality. Cells are classified into red blood cells (HBA1+, HBA2+, HBG1+, HBB+), platelets (PF4+, PPBP+), T cells (CD3D+, CD3E+, CD8A+, CD8B+), and dendritic cells (LYZ+, IFITM3+). Clusters 0, 1, 2, 3, and 4 were defined as T cells (**Figure 3A**). Subsequently, CAR-T cells were distinguished from the T cell population and further analyzed (**Figure 3B**). Based on the characteristics of each subpopulation (**Figure 3C**), CAR-T cells were classified into CD4+Tem (CD4+, CCR7+, GZMB+), CD4+Tn (CD4+, TCF1+, LEF1+), CD8+Teff (CD8A+, CD8B+, GZMB+, GNLY+), CD8+ Tn (CD8A+, CD8B+, TCF1+, LEF1+), and Treg (IL2RA+, FOXP3+). We found that CD4+Tn cells were dominant before reinfusion, while CD4+Tem cells were predominant during the peak of expansion (**Figure 3D**). Compared to the pre-reinfusion state, CD4+ cells at the peak of expansion showed upregulation of cell killing-related genes (NKG7, LAG3, PRF1, CST7, GZMB, GNLY) and memory T cell-related genes (GZMK) (*P* < 0.01), indicating that CAR-T cells at the peak of expansion had stronger killing function and increased memory-like function compared to the pre-reinfusion state (**Figure 3E**; **Supplementary Figure S1**). In addition, both the reinfusion product and the peak of expansion were dominated by CD4+ CAR-T cells. GO functional enrichment analysis of differentially expressed genes revealed upregulation of related pathways such as “inflammatory response”,”cellular response to type II interferon” and “positive regulation of type II interferon production”, which may be associated with the severe CRS observed in patient 4 (**Supplementary Figure S2**) (17).

## Discussion

This study is the first to analyze the efficacy and safety of donor-derived.

CLL-1 CAR-T cells in patients with AML who have relapsed after HSCT. Relapse remains the primary cause of treatment failure in AML. Patients who experience relapse after HSCT often have a poorer prognosis and limited treatment options. In the past, we have found that CLL-1 CAR-T cells therapy is safe and effective in treating relapse and refractory AML. This study further analyzes its efficacy and safety in patients who have experienced relapse after HSCT. The patients included in our study were all classified as extremely high-risk or relapsed multiple times. Despite receiving different dosages of CAR-T cells, all patients exhibited a decrease in tumor burden, with 9 patients achieving CR. CLL-1 CAR-T cells provide an opportunity for remission in patients with very high-risk AML.

Consistent with previous studies, we found that all patients experienced severe cytopenia. Notably, 91.67% of our patients developed grade 4 neutropenia, possibly due to the high expression of CLL-1 in neutrophils. It seems that patients who have undergone transplantation are more prone to severe cytopenia, which may be related to the poor bone marrow proliferative capacity. Fortunately, we discovered that bridging HSCT may compensate for this deficiency. It is well-known that the high treatment-related mortality rate associated with second HSCT contributes to the poor prognosis of AML patients (18). In our study, we opted for a reduced-intensity conditioning regimen to minimize the side effects of second transplant. Due to the myelosuppression, normal engraftment of granulocytes and platelets was achieved even with the reduced-intensity conditioning (19). 7 patients have a better prognosis after CLL-1 CAR-T cells and second transplant. There are even studies showing that bridging HSCT directly after CAR-T cells therapy, without any preconditioning (20).

Given that stem cell infusion from donors can treat persistent pancytopenia after CD19 CAR-T cells therapy with minimal side effects, 2 patients with poor baseline conditions received donor-derived stem cells after CLL-1 CAR-T cells. Unfortunately, both were died because of severe CRS or severe infection. The effectiveness and safety of donor stem cell infusion following CLL-1 CAR-T cells require further investigation. Additionally, some studies have shown that infusion of CD34-positive stem cells can also treat long-term cytopenia after CAR-T cells (21). These approaches may potentially shorten the duration of long-term cytopenia and reduce treatment-related mortality at the same time.

Furthermore, scRNA-seq was used in a CAR-T cells infusion products and PBMCs at the peak of expansion for patient 4, who were acquired MRD-CR but died of severe CRS. We found that both the reinfusion product and the peak of expansion were dominated by CD4+ CAR-T cells. So we speculate that a higher number of CD4 CAR-T cells may be associated with severe CRS. Thus, adjusting the CD4:CD8 CAR-T cell ratio prior to CART cell infusion may help mitigate CAR-T cell-related side effects.

In summary, our study suggests that CLL-1 CAR-T cells, as a novel immunotherapy approach, may be a viable option for patients with relapsed AML after HSCT, particularly for those who have failed traditional treatment methods. However, CLL-1 CAR-T cells may lead to severe neutropenia, bridging to a second transplantation or infusion of donor-derived stem cells may shorten the duration of neutropenia and extend patient survival.

## Conclusions

The allogeneic-derived CLL-1 CAR-T cells therapy exhibits both safety and efficacy in the management of relapsed AML subsequent HSCT. And adjusting the ratio of CD4+ CAR-T cells and CD8+ CAR-T cells prior to infusion may help mitigate CAR-T cell-related side effects.

## Data Availability

The original contributions presented in the study are included in the article/**Supplementary Material**. Further inquiries can be directed to the corresponding author.

## Supplementary material

Supplementary Figure 1 Expression of differentially regulated genes in CAR^pos^ T cells pre- and post-infusion, *P*<0.01.

Supplementary Figure 2 Bubble diagram shows the results of KEGG enrichment analysis between pre- and post-CAR-T infusion.
