# Supplementary figures and images for: Utilization of donor CLL-1 CAR-T cells for the treatment of relapsed of acute myeloid leukemia following allogeneic hematopoietic stem cell transplantation
Source: Front Immunol. 2025 Jan 17;15:1491341. doi: 10.3389/fimmu.2024.1491341 (PMC11782272; doi:10.3389/fimmu.2024.1491341)

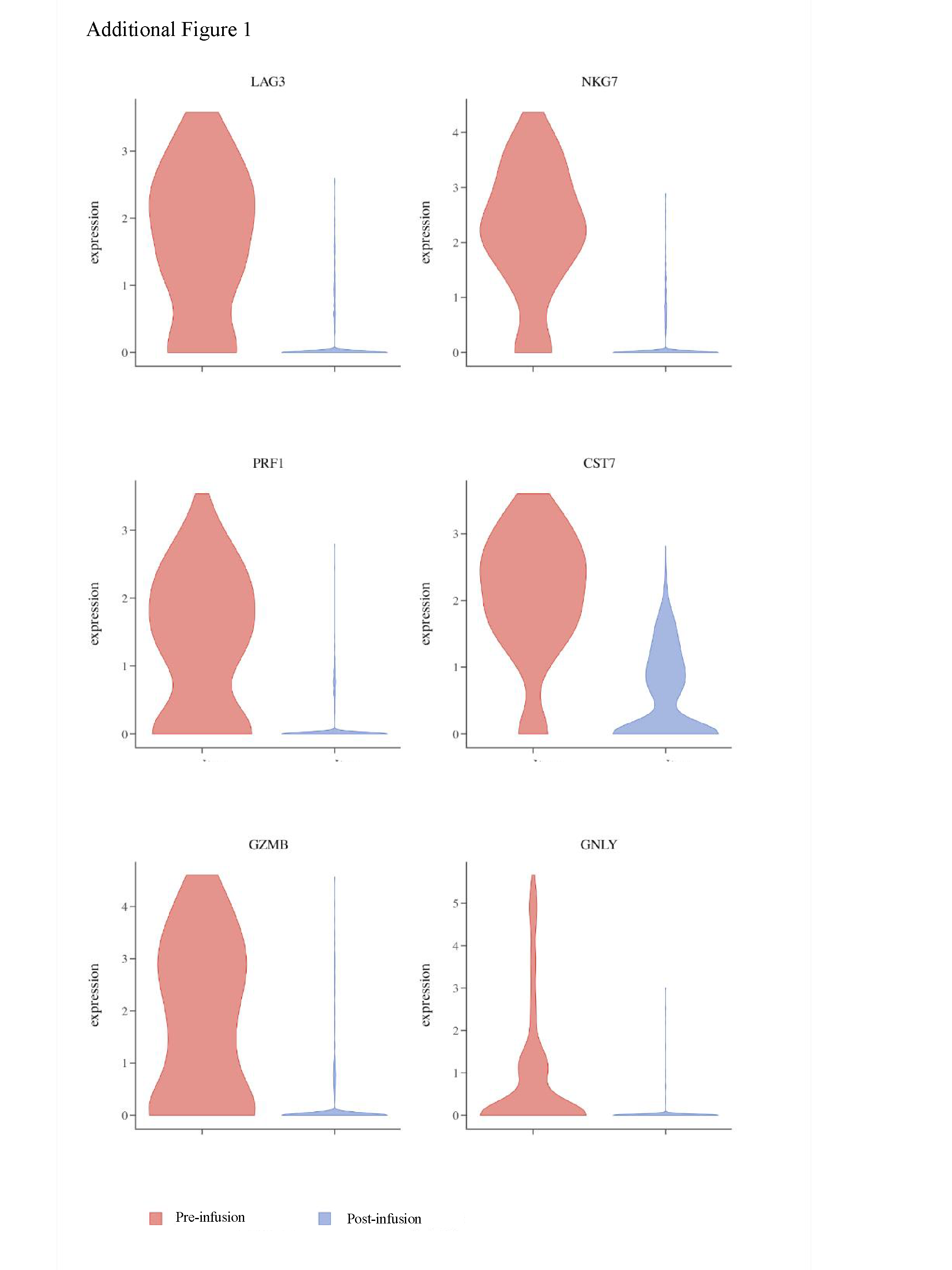

Supplement: Supplementary Figure 1 — Expression of differentially regulated genes in CARpos T cells pre- and post-infusion, P<0.01. [file Image1.tif]

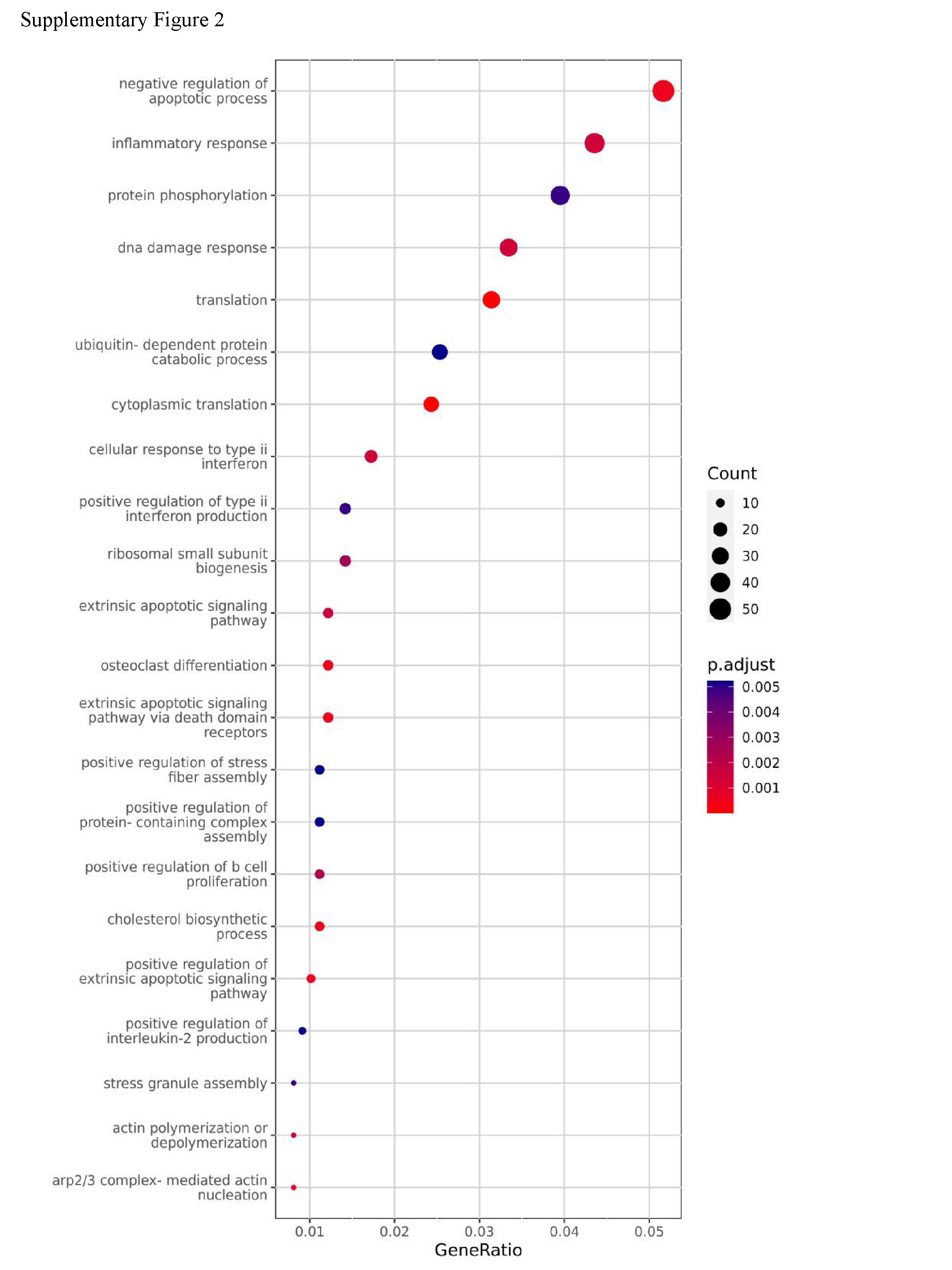

Supplement: Supplementary Figure 2 — Bubble diagram shows the results of KEGG enrichment analysis between pre- and post-CAR-T infusion. [file Image2.tif]
